# Trimethylamine N-oxide (TMAO) disrupts endothelial junction integrity through VE-cadherin Tyr658 phosphorylation in vitro

**DOI:** 10.1007/s00204-026-04368-1

**Published:** 2026-03-31

**Authors:** Carolina Amaral Bueno Azevedo, Guilherme Miniskiskosky, Maria Vitória Guimarães del Amo, Vitória Povala Theisen, Célia Regina Cavichiolo Franco, Regiane Stafim da Cunha, Andréa Emilia Marques Stinghen

**Affiliations:** 1 Experimental Nephrology Laboratory, Basic Pathology Department, Universidade Federal do Paraná, Curitiba, Brazil; 2 Cell Biology Department, Universidade Federal do Paraná, Curitiba, Brazil

**Keywords:** Uremic toxins, Cardiovascular disease, Chronic kidney disease, TMAO

## Abstract

Endothelial dysfunction induced by the accumulation of uremic toxins is a key contributor to cardiovascular disease (CVD) in patients with chronic kidney disease (CKD), as increased endothelial permeability represents an early and critical event in vascular injury and disease progression. This study evaluated the effects of trimethylamine N-oxide (TMAO), a low-molecular-weight uremic toxin, on endothelial junction integrity. Endothelial cells (EA.hy 926) were treated with TMAO at normal (2.83 mg/L) and uremic (7.49 mg/L) concentrations for 24 h. Cell viability was assessed using the MTT assay. VE-cadherin expression and phosphorylation were analyzed by western blotting and confirmed by confocal immunofluorescence. Endothelial permeability was determined by FITC–dextran passage across transwell inserts, and cell adhesion to extracellular matrix proteins was evaluated using fibronectin-coated plates. Our results demonstrate that TMAO reduced cell viability and disrupted endothelial cell–cell junctions by decreasing VE-cadherin protein levels and increasing its phosphorylation at tyrosine 658, resulting in increased endothelial permeability. TMAO also reduced endothelial adhesion to fibronectin, a key extracellular matrix protein. Notably, TMAO did not alter the expression of VE-cadherin–associated proteins, including p120-catenin and β-catenin. These findings provide mechanistic insight into the direct toxic effects of TMAO on endothelial barrier function and identify endothelial junctions as potential therapeutic targets to mitigate cardiovascular risk in CKD.

**Supplementary Information:**

The online version contains supplementary material available at 10.1007/s00204-026-04368-1.

## Introduction

Chronic kidney disease (CKD) is a major and growing challenge facing health systems. Data from the Global Burden of Disease (GBD) from 1990 to 2021 indicate that CKD is the 11th leading cause of death and kidney dysfunction is to be the 8th leading cause of disability-adjusted life years (DALYs) (Duncan et al. 2024). CKD is a progressive and multisystemic condition, and patients affected by it face a significantly higher risk of developing cardiovascular disease (CVD), a major contributor to global morbidity and mortality. As CKD advances, the kidneys progressively lose their capacity to properly filter the blood and eliminate harmful solutes, known as uremic toxins. The accumulation of these compounds in the bloodstream results in uremia, a pathological state characterized by metabolic disturbances and systemic complications. (Vanholder et al. 2008; Stinghen and Pecoits-Filho 2011).

Endothelial dysfunction (ED) is a key contributor to the development of atherosclerosis and CVD. ED is a state in which the vascular endothelium loses its normal functions, including the ability to produce sufficient nitric oxide (NO) and regulate vasodilation, maintain antithrombotic and anti-inflammatory properties, and control vascular permeability (Sun et al. 2020; Roumeliotis et al. 2020; Jeon 2021). The accumulation of uremic toxins leads to ED even in early CKD stages and is an independent predictor of cardiovascular morbidity and mortality in this population. Trimethylamine N-oxide (TMAO) is a gut microbiota-derived metabolite with significant implications for both CKD and CVD. Despite being part of the group of uremic toxins easily removed by dialysis, high levels of TMAO, found even in dialysis patients, are associated with the development of cardiovascular disease, long-term mortality and lower long-term survival in patients with CKD (Tang et al. 2015; Stubbs et al. 2016; Kim et al. 2016; Gruppen et al. 2017; Zhou et al. 2022). At uremic concentrations, TMAO-induced vascular damage is characterized by increased expression of inflammatory markers and vascular calcification (Seldin et al. 2016; Sun et al. 2016; Ma et al. 2017; Chen et al. 2017; Ke et al. 2018; Cheng et al. 2019; Yang et al. 2019; Wu et al. 2020; Querio et al. 2022). However, there is limited understanding regarding whether this uremic toxin adversely affects endothelial cell–cell junctions and permeability.

Endothelial cells form continuous monolayers along blood vessels, and their intercellular junctions, including adherent junctions (AJs) and tight junctions (TJs), are critical to vascular barrier integrity, permeability, and leukocyte transmigration. A major AJ protein is vascular endothelial-cadherin (VE-cadherin), which links adjacent cells and connects to the actin cytoskeleton via catenins (β-catenin and p120 catenin) (Komarova et al. 2017; Duong and Vestweber 2020). Our group has demonstrated that, in the context of uremia, endothelial cell‑to‑cell junctions are progressively disrupted, an effect that intensifies with advancing CKD and involves key adhesion molecules such as VE‑cadherin and the tight‑junction protein ZO‑1 (Maciel et al. 2018). In a recent study, we showed that uremic concentrations of TMAO significantly reduce ZO‑1 protein expression in endothelial cells, thereby impairing tight‑junction integrity and suggesting a mechanistic link between uremic milieu and vascular barrier dysfunction (Azevedo et al. 2024).

In this scenario, we aimed to evaluate the effects of TMAO on the expression of VE-Cadherin in endothelial cells adherents’ junctions, to better understand the pathophysiology of TMAO in disruption of intercellular connections and cardiovascular impact.

## Methodology

### Materials

Dulbecco’s Modified Eagle Medium (DMEM), Fetal Bovine Serum (FBS), penicillin/streptomycin were purchased from Gibco (Grand Island, NY, USA). NorthernLights™ 557 conjugated anti-mouse IgG secondary antibody (NL007**)** and Human Fibronectin Coated Microplates were obtained from R&D Systems (Minnesota, MN, USA). Phospho-VE-cadherin (Tyr658) Polyclonal Antibody (PA5-143661), Anti-VE-cadherin Monoclonal Antibody (14-1449-82), Horseradish peroxidase-conjugated goat anti-mouse IgG (31,430), goat anti-rabbit IgG antibodies (31,460), and Alexa Fluor 488 secondary antibody (A-11008) were purchased from ThermoFisher Scientific (Massachusetts, MA, USA). Anti-β-catenin Monoclonal Antibody (8480 T) was purchased from Cell Signalling Technology (Danvers, MA, USA). Trizol (15,596,026), primers, nitrocellulose membranes (88,025), ActinRed™ 555 ReadyProbes™ (R37112), Fluoromount G with DAPI (00-4959-52) were purchased from Invitrogen (Carlsbad, CA, USA). Trimethylamine n-oxide (317,594) was purchased from Sigma-Aldrich (St. Louis, MO, USA).

### Endothelial cell culture and treatment conditions

An immortalized human endothelial cell line EA.hy926 (ATCC CRL 2922, Manassas, VA, USA) was cultured in DMEM supplemented with 10% fetal bovine serum (FBS) and 10 mg/mL of penicillin/streptomycin and maintained at 37 °C in a humidified atmosphere containing 5% CO_2_. The cells were treated with TMAO at normal (2.83 mg/L) and uremic concentrations (7.49 mg/L) according to the European Uremic Toxin Work Group (EuTox- http://eutoxdb.odeesoft.com/index.php) for 24 h.

### MTT cell viability assay

The cell density of each well was of 10^4^. After 24 h, the cells were treated with TMAO for 24 h and then incubated with MTT (3-(4,5-Dimethylthiazol-2-yl)-2,5-diphenyltetrazolium bromide) solution (5 mg/mL) for 4 h. The culture medium was replaced with dimethyl sulfoxide (DMSO), and the absorbance was measured at 570 nm. Six independent experiments were performed in triplicates.

### F-actin staining by fluorescence microscopy

Cells were plated on circular coverslips (7 × 10^4^ cells), treated with TMAO for 24 h and fixed with 2% PFA. To stain F-actin, two drops (aprox. 50 uL) of ActinRed™ 555 ReadyProbes™ were added per milliliter of medium. The cells were them incubated for 30 min, protected from light. The coverslips were mounted on histological slides with Fluoromount G and DAPI. The cells were observed under a Nikon A1RSiMP + confocal fluorescence microscope (Nikon Instruments, Tokyo, Japan). Two independent experiments were performed in duplicates.

### VE-cadherin, β-catenin and p120 gene expression

Total RNA was isolated from the lysed cells using Trizol method. RNA purity and concentration were checked by measuring the A260 nm/A280 nm absorbance ratio on the NanoDrop 2000 spectrophotometer (ThermoFisher Scientific, Massachusetts, MA, USA). The RNA integrity was analyzed by agarose gel (1%) electrophoresis. The mRNA was transcribed into complementary DNA (cDNA) using the High-Capacity RNA-to-cDNA Kit (Applied Biosystems, Foster City, CA, USA). The cDNA was amplified with specific primers (Table 1) and the SYBR™ Green PCR Master Mix (Applied Biosystems, Foster City, CA, USA) using the StepOnePlus™ System (ThermoFisher Scientific, Massachusetts, MA, USA). The relative gene expression was analyzed using the 2-∆∆CT method (Livak and Schmittgen 2001; Bustin et al. 2009; Huggett et al. 2013). The Hypoxanthine Phosphoribosyltransferase (HPRT) was used as a housekeeping gene. Six independent experiments were performed in duplicates.

**Table 1:** Sequence of the specific primers used for gene amplification

Target gene	Primers
VE-cadherin	5′-CAGCCCAAAGTGTGTGAGAA-3′ (F)
	5′-CGGTCAAACTGTCCATACTT-3′ (R)
p120	5′-GATGCTGTCAAGTCCAATGCAG-3′ (F)
	5′-AGTACTGGGATGCCCTTGAGC-3′ (R)
β-catenin	5′-GTGCTATCTGTCTGCTCTAGTA-3′ (F)
	5′-CTTCCTGTTTAGTTGCAGCATC-3′ (R)
HPRT	5′-GAACGTCTTGCTCGAGATGTGA-3′ (F)
	5′-TCCAGCAGGTCAGCAAAGAAT-3′ (R)

*F* Forward, *R* Reverse

### VE-cadherin western blot analysis

Approximately 3 × 10^6^ cells were washed with ice-cold PBS and lysed in 100 µL of radioimmunoprecipitation assay buffer (150 mM sodium chloride, 1.0% Triton X-100, 0.5% sodium deoxycholate, 0.1% SDS (sodium dodecyl sulfate), 50 mM Tris, pH 8.0) for 20 min. The total protein concentration was performed using Bradford assay. Equal amounts of protein (25 µg) were separated using 7% SDS-PAGE and transferred onto nitrocellulose membranes. The membranes were blocked for 1 h in Tris-buffered saline containing 5% BSA and 0.3% Tween 20. After washing with TBS-Tween 20 (0.05%), the membranes were incubated overnight with 1 µg/mL of rabbit anti-human Phospho-VE-cadherin (Tyr658) Polyclonal Antibody at 4 ◦C. Primary antibodies were detected using a horseradish peroxidase conjugated-goat anti-rabbit IgG (0.01 mg/mL) antibody and visualized by SuperSignal™ West Femto (ThermoFisher Scientific). After the detection of Phospho-VE-cadherin, the membranes were stripped using a strip solution, blocked with for 1 h in Tris-buffered saline containing 5% BSA and 0.3% Tween 20 and incubated overnight with 1µg/mL of mouse anti-human VE-cadherin at 4 °C. Primary antibodies were detected using a horseradish peroxidase conjugated-goat anti-mouse IgG (0.01 mg/mL) antibody and protein bands were visualized using by SuperSignal™ West Femto (ThermoFisher Scientific). Band intensities were quantified by densitometry and normalized to β-actin. Band intensity was analyzed using the Software Image Studio Lite 5.0 (Lincoln, NE, USA). Six independent experiments were performed in triplicates.

### β-catenin western blotting analysis

For β-Catenin analysis, a nuclear and cytoplasmatic protein extraction were performed. Briefly, approximately 3 × 10^6^ cells were washed with ice-cold PBS and collected with the help of a cell scrapper. Cells were centrifuged for 3 min at 1.500 rpm and the pellet were resuspended gently with 500 µL of 1 × hypotonic buffer (20 mM Tris–HCl (pH 7.4), 10 mM NaCl, 3 mM MgCl_2_). After 20 min, 25µL of 10% IGEPAL® CA-630 (Sigma-Aldrich) detergent was added. The homogenate was centrifuged for 10 min at 3.000 rpm and the supernatant containing the cytoplasmatic fraction was stored at − 80 °C. The pellet was resuspended in 50 µL of radioimmunoprecipitation assay buffer (150 mM sodium chloride, 1.0% Triton X-100, 0.5% sodium deoxycholate, 0.1% SDS (sodium dodecyl sulfate), 50 mM Tris, pH 8.0), incubated on ice for 30 min with vortexing at 10-min intervals and then centrifuged for 30 min at 13.000 rpm. The supernatant containing the nuclear fraction was stored at − 80 °C. The protein concentration was performed using Bradford assay. Equal amounts of protein (20 µg) were separated using 8% SDS-PAGE and transferred onto nitrocellulose membranes. The membranes were blocked for 1 h in Tris-buffered saline containing 5% casein and 0.3% Tween 20. After washing with TBS-Tween 20 (0.05%), the membranes were incubated overnight with 1 µg/mL of rabbit anti-human β-Catenin Polyclonal Antibody (Cell Signaling) at 4 ◦C. Primary antibodies were detected using a horseradish peroxidase conjugated-goat anti-rabbit IgG (0.01 mg/mL) antibody and protein bands were visualized using by SuperSignal™ West Femto (ThermoFisher Scientific). Band intensities were quantified by densitometry. Nuclear fractions were normalized to Lamin-A and cytoplasmatic fractions were normalized to β-actin. Band intensity was analyzed using the Software Image Studio Lite 5.0 (Lincoln, NE, USA). Three independent experiments were performed in triplicates.

### VE-cadherin immunofluorescence analysis

7 × 10^4^ cells were plated on circular coverslips, treated, and fixed with 2% PFA, followed by washing with PBS and incubation with 0.1% glycine. Subsequently, the cells were incubated with PBS containing 1% BSA and 0.01% saponin for 1 h. The cells were then incubated with 0.5 µg/mL of an anti-human Phospho-VE-cadherin (Tyr658) Polyclonal Antibody in humid chamber overnight at 4 °C. The coverslips were then washed and incubated with anti-human VE-cadherin antibody in humid chamber overnight at 4 °C overnight. The coverslips were then washed again and incubated with NorthernLights™ 557 conjugated anti-mouse IgG secondary antibody (1 mg/mL) for 1 h. The coverslips were mounted on histological slides with Fluoromount G and DAPI, sealed with formalin-free colorless enamel and observed under a Nikon A1RSiMP + confocal fluorescence microscope (Nikon Instruments, Tokyo, Japan). Three independent experiments were performed in duplicates.

### Cell permeability assay

Approximately 2 × 10^5^ cells were plated per well on the top of Transwell® inserts and incubated at 37 ◦C until confluence. The cells were then treated for 24 h, washed and a FITC-Dextran solution (2.5 mg/mL) was added to each insert. After 20 min in the absence of light, an aliquot from the lower compartment was transferred to a 96-well black microplate and the fluorescence was read with Tecan Infinite® 200 PRO microplate reader (Tecan Group Ltd., Männedorf, Switzerland) (λexc = 485 nm and λem 535 nm). Data were analyzed using the Tecan i-control software Version 1.5.14.0 (Tecan, Salzburg, Austria, 2008). The insert membranes were then stained with crystal violet for further visualization under an inverted microscope (MOTIC AE2000 with MOTICAM S6). The results were expressed as % control (non-treated cells). Three independent experiments were performed in triplicates.

### Cell adhesion assay

Approximately 1.5 × 10^6^ cells were plated on tissue culture dishes with 10 cm diameter. After 24h cells were treated for 24h. Cells were then detached with EDTA, washed with PBS and 10^4^ cells were plated on each well of Human Fibronectin Coated Plate (R&D). The plate was incubated at 37 °C for 2 h. After, each well was washed to eliminate non-attached cells and fixed with methanol for 10 min. Cells were stained with violet crystal for 20 min, washed 6 times with PBS and photos of each well were taken with an inverted microscope (MOTIC AE2000 with MOTICAM S6). After, the violet crystal was eluted with 33% of glacial acetic acid and the absorbance was measured at 540 nm. Three independent experiments were performed in triplicates.

### Statistical analysis

The Shapiro–Wilk normality test was used, followed by the Student’s t-test or Anova test to statistically analyze parametric data. For non-parametric data, a Mann–Whitney or Anova on Rank’s tests was used. A *P* < 0.05 value between groups was considered significant. Values were expressed as means ± standard error of the mean (SEM). Data were analyzed using the GraphPad Prism 6.01 statistical package (GraphPad Software Inc., La Jolla, CA, USA, 2012).

## Results

### TMAO reduces cell viability and modifies the endothelial cell cytoskeleton

**Fig. 1:**
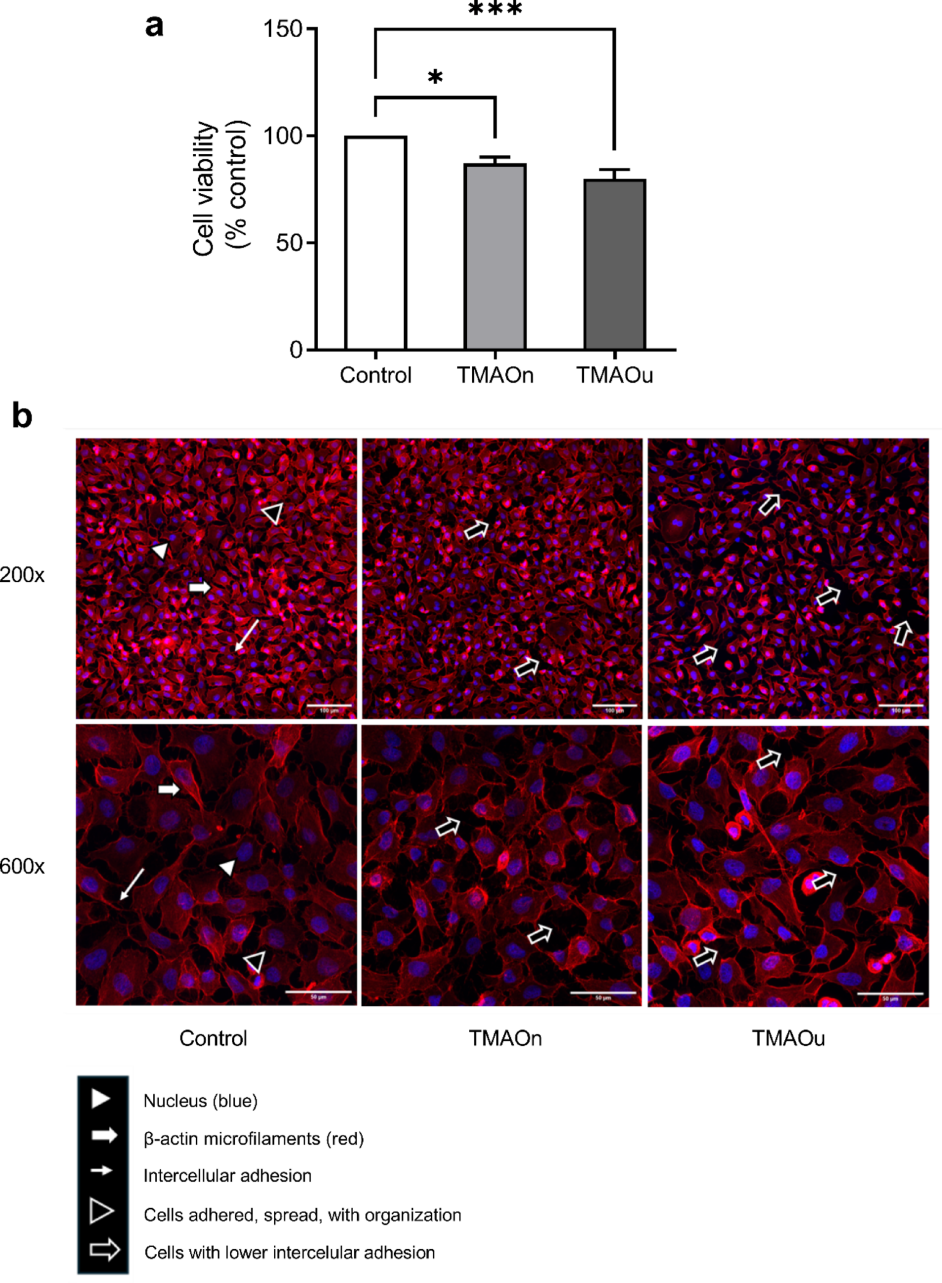
**a** Effect of TMAO on cell viability. Control (non-treated cells), TMAOn (TMAO at normal concentrations), TMAOu (TMAO at uremic concentrations). Cells were incubated with normal (2.83 mg/L) and uremic (7.49 mg/L) concentrations of TMAO for 24h. Cell viability was assessed by MTT (3-(4,5-Dimethylthiazol-2-yl)-2,5-diphenyltetrazolium bromide) method, n = 18. Results are expressed as % of control (non-treated) cells. *P < 0,05: TMAOn; ***P < 0,001: TMAOu. **b** Effect of TMAO on F-actin protein organization. After treatment with normal (TMAOn) and uremic (TMAOu) concentrations, F-actin protein organization was visualized using ActinRed (red). DAPI (4’,6-diamidino-2-phenylindole) was used to label the nuclei (blue). Control (non treated cells), magnification 200× and 600×

Cell viability significantly decreased in cells treated with TMAO at both, normal and uremic concentrations when compared to control (non-treated cells) (Fig. 1a). The association of actin cytoskeleton and cell adhesion proteins is fundamental in the organization of cell junctions and barrier function. Cells treated with TMAO at normal concentrations still maintained a pattern similar to that of control cells, with cells remained adhered and dispersed, exhibiting a central nucleus, although there is a slight decrease in intercellular adhesion. Cells treated with uremic concentrations showed fewer cells reduced intercellular adhesion (Fig. 1b).

### TMAO impacts cell-to-cell junctions trough diminishing VE-Cadherin Expression and increasing VE-Cadherin phosphorylation

**Fig. 2:**
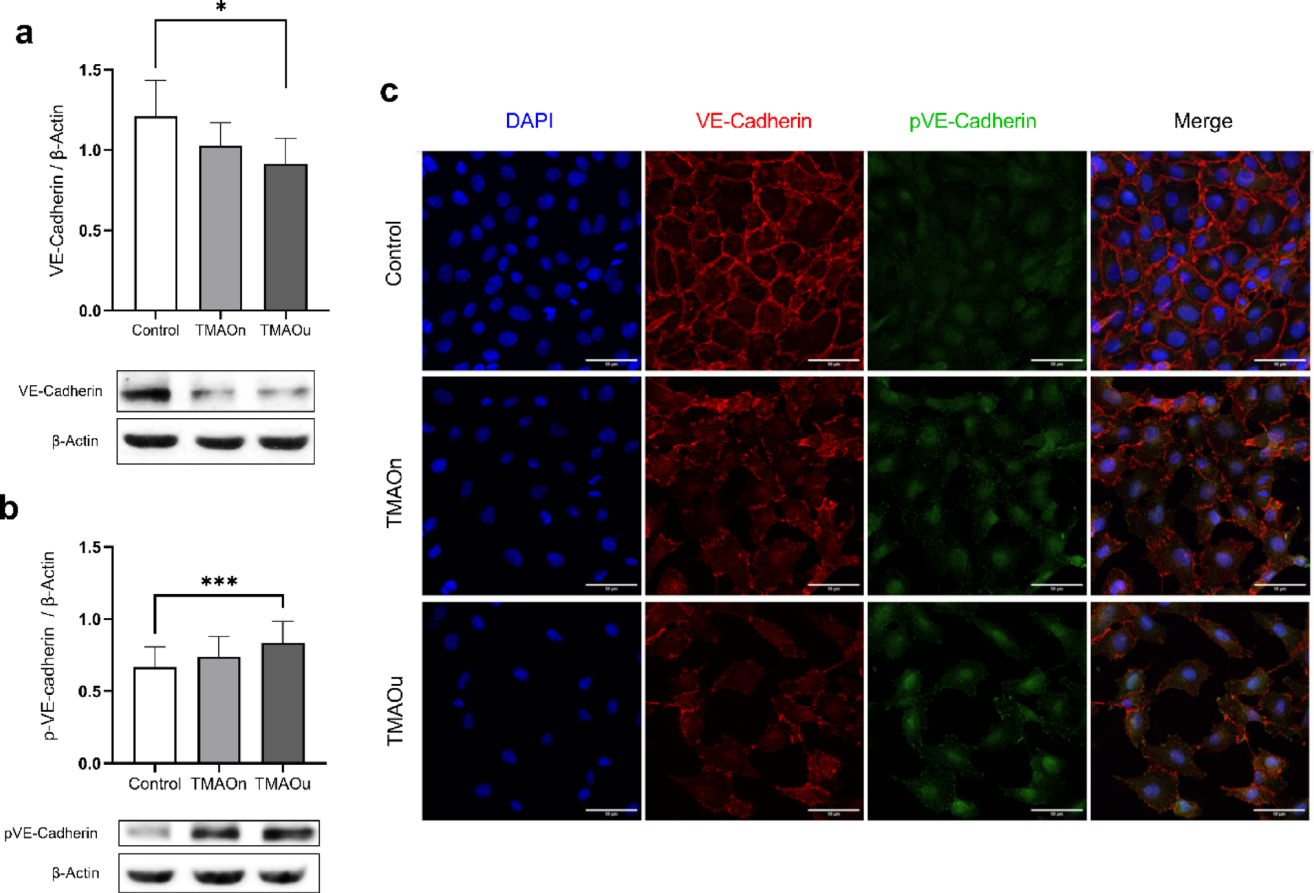
TMAO impacts endothelial cells levels of VE-cadherin and its phosphorylation. Control (non-treated cells), TMAOn (TMAO at normal concentrations), TMAOu (TMAO at uremic concentrations). Effects of TMAO on VE-Cadherin protein expression by immunoblotting: *P* < 0.05 control *vs* uremic (**a**). Effect of TMAO on Phospho-VE-cadherin protein expression by immunoblotting: *P* < 0.0003 control *vs* uremic (**b**). β-actin was used as protein loading control. Lower panel: representative immunoblot. Upper panel: bands quantification. Effects of TMAO on VE-Cadherin protein expression and its phosphorylation by immunofluorescence staining (**c**). Magnification 600x

We investigated the gene expression of VE-Cadherin, and no significant differences were found (Figure S1a). Regarding protein expression, we found a significant decrease in VE-Cadherin levels (Fig. 2a; *P* < 0.05) and a significant increase in its phosphorylated (at tyrosine 658) form (Fig. 2b; *P* < 0.0003) when endothelial cells were treated with uremic concentrations of TMAO compared to control (non-treated cells). To assess the localization of VE-Cadherin and its phosphorylated form, we performed immunofluorescence staining (Fig. 2c). In cells treated with TMAO at uremic concentrations a decrease in VE-cadherin and an increase in phospho-VE-cadherin were observed, corroborating the Western blot data. Collectively, these results demonstrate that TMAO has a significant impact on VE-cadherin protein levels and is associated with its increased phosphorylation. To assess whether TMAO also influences VE-cadherin–associated proteins, we evaluated the gene expression of p120-catenin (Figure S1b) and β-catenin (Figure S1c), but no significant differences were observed. Given its role in nuclear signaling, β-catenin was further analyzed in cytoplasmic and nuclear fractions, which also showed no significant differences (Figure S2). Thus, TMAO appears to selectively modulate VE-cadherin without affecting its associated catenins.

### TMAO increases endothelial cell permeability

**Fig. 3:**
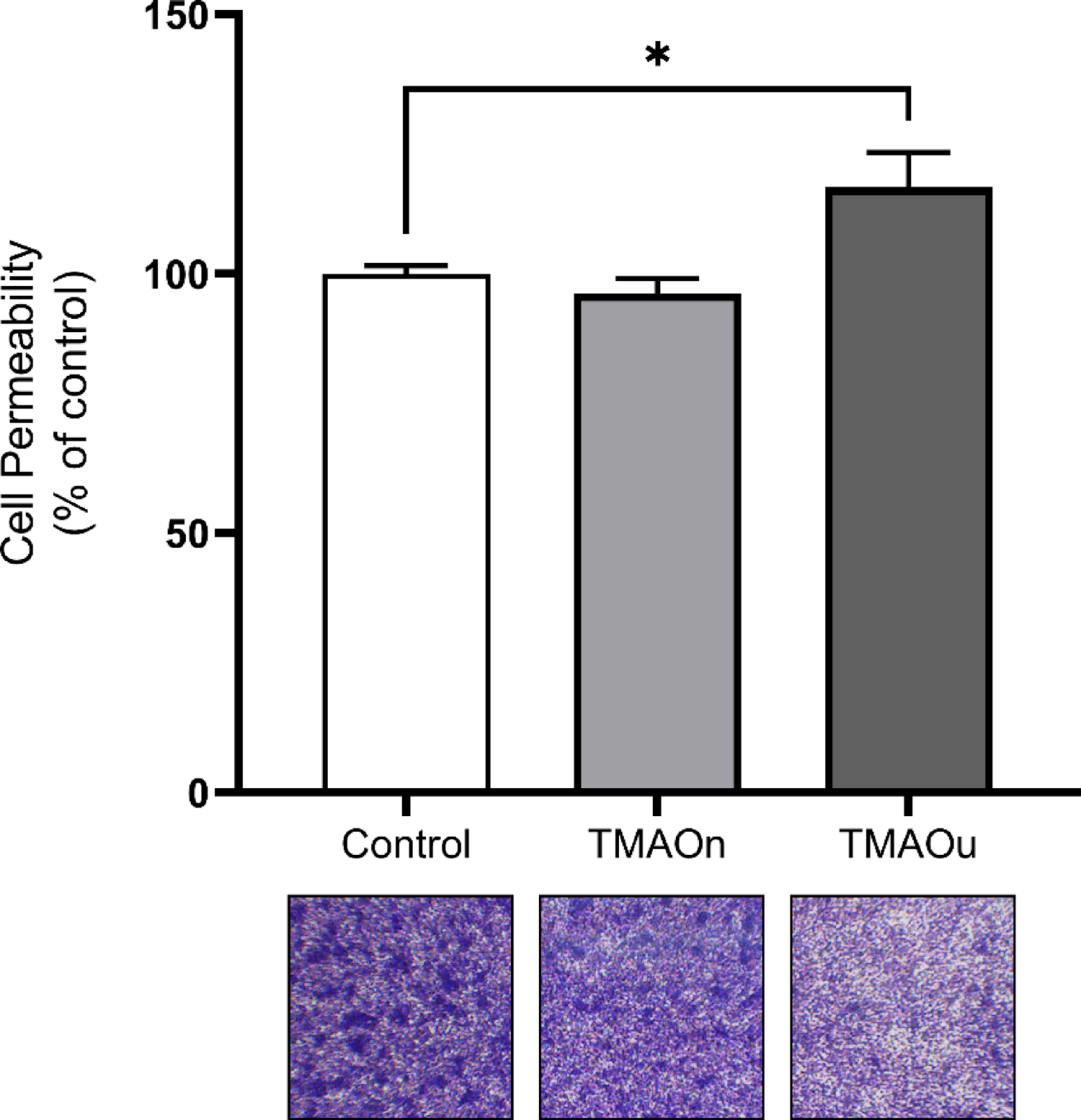
TMAO effect on cell permeability. Control (non-treated cells), TMAOn (TMAO at normal concentrations), TMAOu (TMAO at uremic concentrations). Cells were incubated with TMAO at normal (2.83 mg/L) and uremic (7.49 mg/L) concentrations for 24 h. Paracellular permeability was assessed by measuring the passage of Fluorescein isothiocyanate (FITC)-dextran through the endothelial monolayer, n = 8. Results are expressed in % of control (non-treated) cells. *P < 0,05: TMAOu

Cell permeability was evaluated after exposed to TMAO at normal and uremic concentrations. There was a significant increase in endothelial permeability of cells treated with uremic concentrations of TMAO (*P* < 0.05) compared with untreated control (non-treated) cells (Fig. 3). These results were further confirmed by visualization of the endothelial monolayer on the transwell inserts after staining with crystal violet (Fig. 3, lower panel).

### TMAO reduces endothelial cell adhesion to fibronectin

**Fig. 4:**
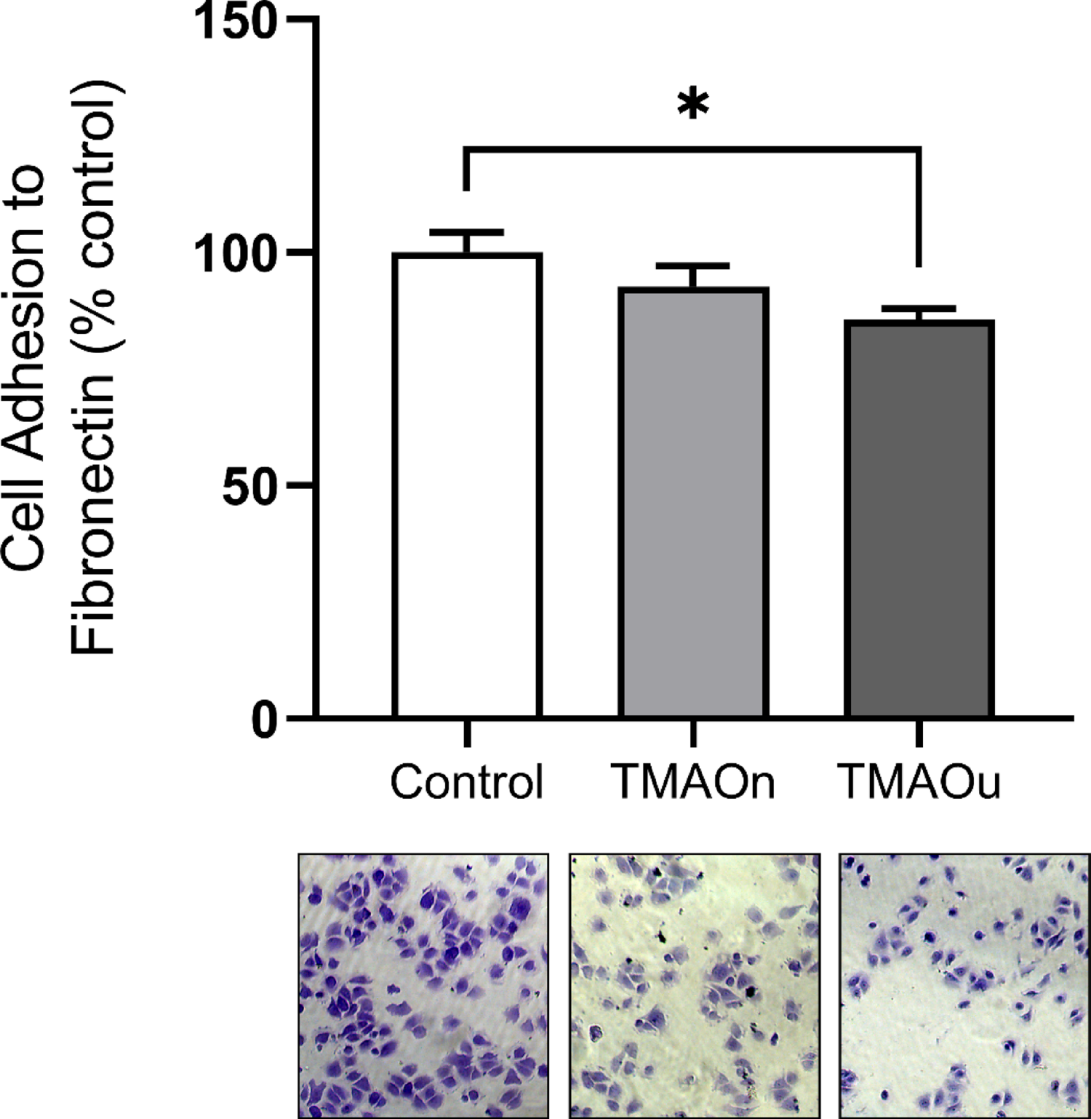
TMAO effect on cell adhesion to fibronectin. Control (non-treated cells), TMAOn (TMAO at normal concentrations), TMAOu (TMAO at uremic concentrations). Cells were incubated with TMAO at normal (2.83 mg/L) and uremic (7.49 mg/L) concentrations for 24 h. Cell adhesion was assessed by violet crystal method with human fibronectin coated microplates (R&D Systems), n = 9. Results are expressed as % of control (non-treated) cells. *P < 0,05: TMAOu

Cell adhesion to fibronectin was significantly reduced in cells treated with TMAO at uremic concentrations (*P* < 0.05) when compared to untreated control (non-treated) cells (Fig. 4). We also analysed vitronectin and laminin, but no significant differences were found (data not show).

## Discussion

Endothelial dysfunction caused by the accumulation of uremic toxins is an important contributor to the development of cardiovascular disease (CVD) in patients with chronic kidney disease (CKD). This study evaluated the impact of the uremic toxin trimethylamine N-oxide (TMAO) on endothelial morphology and cell junction proteins. The main findings of the present study are as follows: (1) at uremic concentrations TMAO significantly reduces endothelial cell viability; (2) TMAO modifies endothelial cell cytoskeleton and decreases intercellular adhesions; (3) TMAO impacts endothelial cell-to-cell junctions through a decrease in VE-cadherin protein expression and an increase in its phosphorylation at tyrosine 658, thus increasing endothelial cell permeability; (4) TMAO reduces endothelial cell adhesion to fibronectin. To our knowledge, this is the first study to demonstrate the effect of TMAO on endothelial adherent junctions and endothelial permeability.

TMAO has been shown to be strongly associated with decreased glomerular filtration rate (GFR), which leads to worsening of CKD and endothelial dysfunction, activating important inflammatory pathways and increasing the risk of cardiovascular events and mortality in patients with CKD (Tang et al. 2015; Stubbs et al. 2016; Kim et al. 2016; Gruppen et al. 2017; Xu et al. 2017; Bir Singh et al. 2019; Cheng et al. 2019; Pelletier et al. 2019). Our data demonstrate that TMAO was able to significantly reduce endothelial cell viability of human endothelial cells exposed to normal (2.83mg/L) and uremic (7.49mg/L) concentrations.

Endothelial adherent junctions maintain endothelial integrity, its barrier function and mediates endothelial permeability, regulating the trafficking of molecules between circulation and tissue. The disruption of endothelial cell-to-cell junctions and increase in endothelial permeability contribute to progression of many pathologies, including cardiovascular diseases (Claesson-Welsh et al. 2021). Studies showed that uremic toxins (*p*-cresyl sulfate, inorganic phosphate, indoxyl sulfate and uremic serum from patients with CKD) can affect cell-to-cell junctions and increase vascular permeability (Peng et al. 2012; Maciel et al. 2018; Chen et al. 2020). Our data showed that TMAO decreases VE-cadherin levels and increases its phosphorylation at tyrosine 658. Tyrosine phosphorylation of VE-cadherin is associated with the disruption of endothelial cell-to-cell junctions (Mehta and Malik 2006a, b; Kumar et al. 2009; Dejana et al. 2009; Monaghan-Benson and Burridge 2013; Benn et al. 2016; Park-Windhol and D’Amore 2016; Hahn et al. 2024) and can prevent the binding of p120 catenin (p120) and β-catenin to VE-cadherin, which are important accessory molecules that regulate the association of cadherins with the actin cytoskeleton and are essential to strong cell-to-cell adhesion (Potter et al. 2005). Although endothelial barrier function involves multiple intercellular adhesion systems and other proteins, the disruption of VE-cadherin is sufficient to disorganize all other intercellular junctions (Corada et al. 1999; Shen et al. 2011). We also investigated whether TMAO could also affect p120 catenin and β-catenin, but no significant differences were found. Taken together, these observations suggest that the increase in endothelial permeability is driven, at least in part, by TMAO-mediated disruption of VE-cadherin, thereby weakening adherent junction integrity and compromising vascular barrier function.

The adhesion of endothelial cells to extracellular matrix molecules was investigated. Our data demonstrate a decrease in endothelial cells adhesion to fibronectin. The adhesion of endothelial cells to molecules of extracellular matrix is important to maintain the vascular integrity and regulates migration, proliferation and cellular signing. Fibronectin is a major constituent of the basal lamina of the extracellular matrix and its binding to endothelial cells occurs through integrins, mainly integrin α5β1 and αvβ3 (Post et al. 2019). We hypothesize that the decrease in endothelial cells adhesion to fibronectin is related to TMAO effects on integrin α5β1, as we did not observe any difference in adhesion to other molecules of the extracellular matrix, but further experiments are required to be done to confirm this hypothesis.

We recognize that our study has limitations, and further work are required. First, we did not evaluate the effect of TMAO on VE-cadherin internalization after phosphorylation. Second, although informative, in vitro experiments do not fully reproduce in vivo conditions. It has been demonstrated that hemodynamic forces and shear stress can cause VE-cadherin phosphorylation and modulates its regulation (Orsenigo et al. 2012; Conway et al. 2017; Caolo et al. 2018). Our study opens news perspectives to evaluate the involvement of intercellular junction proteins to better elucidate the relationship of specific uremic toxins, as demonstrated here with TMAO, with changes in endothelial injury and endothelial injury and vascular permeability.

## Conclusion

In conclusion, this study provides the first evidence that TMAO directly compromises endothelial barrier integrity by modulating VE-cadherin expression and phosphorylation, thereby weakening intercellular junctions and promoting endothelial vascular permeability. Importantly, uremic concentrations of TMAO significantly reduce endothelial adhesion, suggesting that the progressive accumulation of this metabolite during CKD may accelerate endothelial dysfunction and contribute to the heightened cardiovascular risk observed in these patients.

## Supplementary Information

Below is the link to the electronic supplementary material.

Supplementary Material 1.
