# Supplementary material for: Trimethylamine N-oxide (TMAO) disrupts endothelial junction integrity through VE-cadherin Tyr658 phosphorylation in vitro
Source: Arch Toxicol. 2026 Mar 31;100(7):3063–72. doi: 10.1007/s00204-026-04368-1 (PMC13309513; doi:10.1007/s00204-026-04368-1)
Supplement: Supplementary file 1 — Supplementary Material 1. [file 204_2026_4368_MOESM1_ESM.pdf]

Supplementary Material

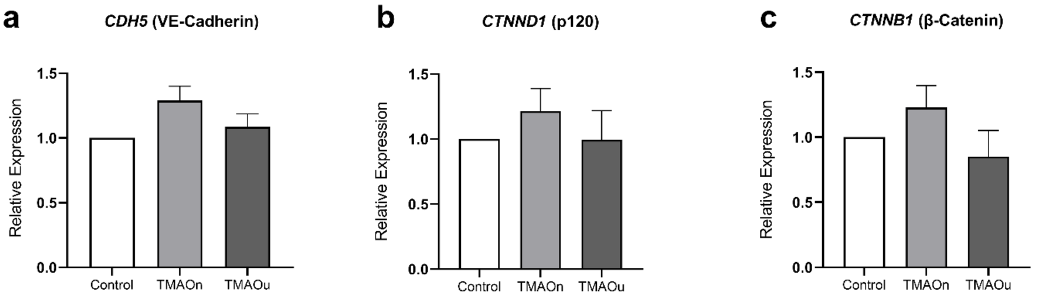

Figure S1. Effect of TMAO on VE-Cadherin (a), p120 (b) and β-catenin (c) gene expression. Control (non-treated cells), TMAOn (TMAO at normal concentrations), TMAOu (TMAO at uremic concentrations). Gene expression was determined by RT-qPCR (n=6). Untreated cells served as the control. Data are expresse as mean ± SEM.

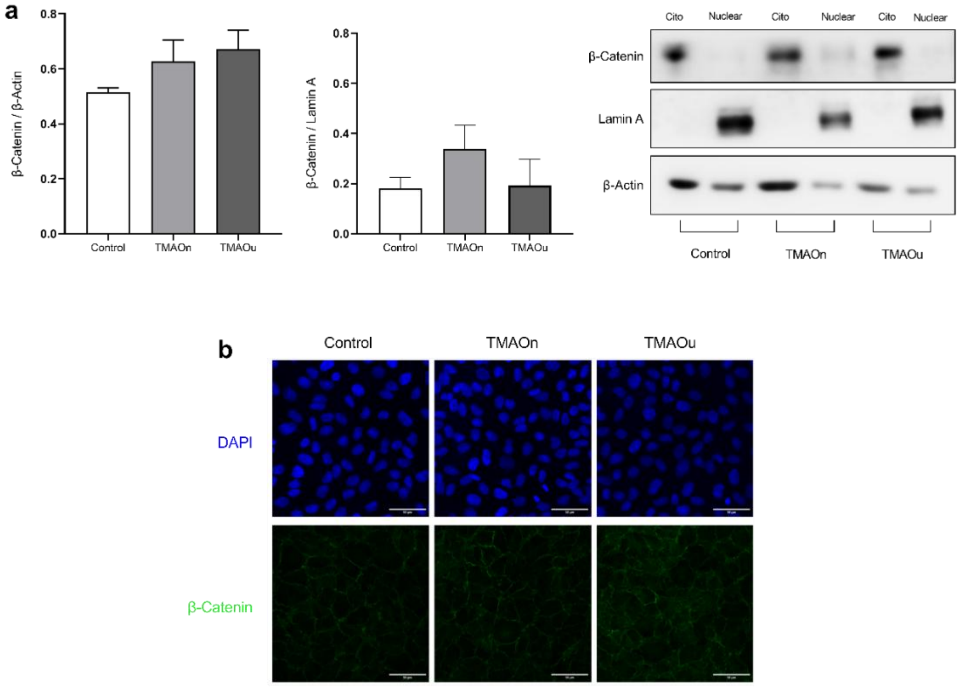

Figure S2. (a) Effect of TMAO on β-catenin protein expression. Control (non-treated cells), TMAOn (TMAO at normal concentrations), TMAOu (TMAO at uremic concentrations). Protein expression was determined by Western blotting (n=9). β-actin was used as the loading control for the cytoplasmic fraction, and Lamin A was used as the loading control for the nuclear fraction. Left panel: band quantification. Right panel: representative immunoblot. (b) Effect of TMAO on β-catenin expression assessed by immunofluorescence staining. Magnification, 600×.
